# Mending the failing heart

**DOI:** 10.18632/aging.101879

**Published:** 2019-03-18

**Authors:** Xiaowei Wang, Karlheinz Peter

**Affiliations:** 1 Atherothrombosis and Vascular Biology Laboratory, Baker Heart and Diabetes Institute Melbourne, Victoria 3004, Australia; 2 Department of Medicine, Monash University, Melbourne, Victoria 3004, Australia

**Keywords:** activated platelets, myocardial infarction, CD39

Acute thrombotic events, such as heart attacks and strokes, are the leading causes of morbidity and mortality worldwide. The heart disease and stroke statistics - 2016 update from the American Heart Association stated that cardiovascular disease (CVD) accounted for 1 in 3 deaths in the United States [1].

One of the major risk factors for developing CVD is aging itself. In most cases of acute heart attack and stroke, the rupture of a vulnerable plaque results in the activation of platelets and the coagulation cascade [2]. Platelets are small anucleate cells and their main function in the body is to maintain hemostasis, a process aimed towards stopping bleeding, as well as to maintain the integrity of the vessel wall in the event of a vascular injury.

The activation of platelets causes the release of dense granules containing large amounts of adenosine triphosphate, serotonin, and the potent platelet agonist adenosine diphosphate (ADP) [3]. This creates a microenvironment with high concentrations of ADP, resulting in the amplification of platelet activation and aggregation [3]. In addition, the activation of platelets switches glycoprotein (GP) IIb-IIIa from an inactive confirmation to an active conformation. This change in conformation results in the ability to cross-link adjacent platelets via fibrinogen, leading to platelet aggregation and the formation of blood clots. Ultimately, this atherothrombotic event occludes the entire vessel, resulting in ischemia and cell death.

Non-invasive anti-platelet and/or anti-thrombotic therapies are used to dissolve the blood clot or to prevent (further) growth of the thrombus, thereby preventing potentially deadly events. However, current anti-platelet drugs inhibit both resting and activated platelets and the use of these drugs have an inherent risk to cause bleeding complications, which hinders their use in the wider community, in particular of elderly patients [3–5]. Even if patients are suffering a heart attack and receive invasive catheterization to reopen the vessel, platelets are known to be contributors to the no flow phenomenon after reperfusion of a blocked coronary artery and therefore have a grimmer prognosis [5].

Our group has developed an alternative therapeutic strategy which blocks the activated GPIIb-IIIa, as well as degrades the potent platelet agonist ADP. By genetic coupling of the ecto-nucleoside triphosphate diphosphohydrolase CD39 to a single-chain antibody (scFv) against the active conformation of GPIIb/IIIa on activated platelets, we have designed a bifunctional molecule termed Targ-CD39 [3,5].

Using Targ-CD39 in a murine model of myocardial ischemia/reperfusion, we have demonstrated improvement of cardiac function via conventional 2-dimension echocardiography using Brightness-mode, as well as reduced cardiac deformation using strain analysis [5]. Echocardiography is extensively used in the clinical setting to investigate the cardiac anatomy, to evaluate its function and to determine the wall motion movements. In the last decade, the technology of strain analysis was incorporated and is now a well-established clinical method for the detection of myocardial deformation. Strain analysis reflects myocardial contractility more accurately than conventional echocardiography, and therefore is a better clinical predictor of mortality as compared to ejection fraction and the wall motion score index [5]. Therefore, we are using the state-of-the-art high frequency ultrasound imaging to perform radial and longitudinal strain analysis for our research, a technique which is relatively novel for small animal ultrasound. Targ-CD39 also reduced infarct size, increased capillary density and deceased microthombi formation in the ischaemic myocardium after reperfusion, thereby preventing further ischaemic tissue damage.

By targeting CD39 to activated GPIIb/IIIa, we can control the amount of drugs required to be injected. This targeting effect homes the drugs to the area of thrombosis, resulting in a maximum therapeutic effect while eliminating the undesirable side effect of bleeding [3,5]. While this study shows a novel and promising treatment in the setting of a heart attack, this targeted drug may also be ideal for other thrombotic diseases, including strokes, deep vein thrombosis and pulmonary embolism [6].

The ability to target activated GPIIb/IIIa is ideal for drug delivery [7]. ScFvs are a minimal form of a functional antibody, consisting of the variable heavy and variable light chain where the CDR regions reside. They lack the Fc region and therefore are minimally immunogenic [2]. They can be genetically designed with specific tags for detection, specific groups for bioconjugation [2], and as an antibody-drug fusion construct for targeted drug delivery [3,5]. In addition to the anti-inflammatory properties, of CD39, our group has genetically fused the single-chain antibody that targets activated GPIIb/IIIa to several drugs, such as an anti-fibrinolytic single-chain urokinase plasminogen activator [4] and an anti-platelet tick coagulation peptide [8]. These approaches offer similar benefits of thrombus breakdown and/or prevention without any bleeding complications.

Overall, this proof-of-concept study justifies further development towards targeted drug delivery in thrombotic diseases after further extensive experimental testing. Targ-CD39 has the potential to be translated to clinical application and holds promise to be beneficial to many patients with cardiovascular diseases. In particular, elderly patients who have both an increased risk of a myocardial infarction and stroke as well as an increased risk of bleeding complications, may benefit substantially.
